# Dietary supplementation of *Astragalus* flavonoids regulates intestinal immunology and the gut microbiota to improve growth performance and intestinal health in weaned piglets

**DOI:** 10.3389/fimmu.2024.1459342

**Published:** 2024-10-02

**Authors:** Yuyan Che, Lu Li, Mengjie Kong, Yiwen Geng, Dong Wang, Bin Li, Lufang Deng, Guoshun Chen, Jing Wang

**Affiliations:** ^1^ Institute of Animal Husbandry and Veterinary Medicine, Beijing Academy of Agriculture and Forestry Sciences, Beijing, China; ^2^ College of Animal Science and Technology, Gansu Agricultural University, Lanzhou, China; ^3^ Sino-US Joint Laboratory of Animal Science, Beijing Academy of Agriculture and Forestry Sciences, Beijing, China; ^4^ College of Life Science and Food Engineering, Hebei University of Engineering, Handan, China; ^5^ Intelligent Equipment Research Center, Beijing Academy of Agriculture and Forestry Sciences, Beijing, China; ^6^ Department of Technology, Feed Branch of Beijing Sanyuan Breeding Technology Co., Ltd, Beijing, China

**Keywords:** *Astragalus*, flavonoids, weaned piglet, intestinal immune, growth performance

## Abstract

*Astragali Radix* (AS) is a widely used herb in traditional Chinese medicine, with calycosin as its main isoflavonoid. Our previous study discovered that calycosin triggers host defense peptide (HDP) production in IPEC-J2 cells. The aim of this study is to investigate the alleviation effects of AS total flavone and AS calycosin on growth performance, intestinal immunity, and microflora in weaned piglets. Sixty-four piglets were assigned randomly to 4 treatment groups, (1) CON: the basal diet, (2) P-CON: the basal diet plus antibiotics (1 g/kg), (3) AS-TF: the basal diet plus AS total flavone at 60 mg/day per piglet, (4) AS-CA: the basal diet plus AS calycosin at 30 mg/day per piglet. Each treatment consists of 4 replicates with 4 piglets per replicate. Results showed that treatment with AS-TF and AS-CA enhanced average daily growth and average daily feed intake compared to the CON group (*P* < 0.01), while AS-CA significantly reduced the diarrhea rate (*P* < 0.05). Both AS-TF and AS-CA significantly increased serum immunoglobulin (Ig) A and IgG levels, with AS-CA further boosting intestinal mucosal secretory IgA levels (*P* < 0.05). Histological analysis revealed improvements in the morphology of the jejunum and ileum and goblet cell count by AS-TF and AS-CA (*P* < 0.05). Supplementation of AS-TF and AS-CA promoted the expression of several intestinal HDPs (*P* < 0.05), and the effect of AS-CA was better than that of AS-TF. In addition, the AS-TF and AS-CA regulated jejunal microbial diversity and composition, with certain differential bacteria genera were showing high correlation with serum cytokines and immunoglobulin levels, suggesting that the intestinal flora affected by AS-TF and AS-CA may contribute to host immunity. Overall, AS CA and AS TF all improved growth performance and health, likely by enhancing nutrition digestibility, serum and intestinal immunity, and intestinal microbial composition. They showed the similar beneficial effect, indicating AS CA appears to be a major compound contributing to the effects of AS TF. This study demonstrated the positive effect of AS flavonoids on weaned piglets and provided a scientific reference for the efficient use of AS products.

## 1 Introduction

Weaning piglets experience stress due to environmental, physiological, and social challenges, which induce intestinal and immune system dysfunctions, leading to diarrhea, reduced growth rates, and decreased disease resistance (1, 2). Over the past few decades, antibiotics have been crucial in controlling animal diseases and promoting animal growth and immune system function by boosting immunity and promoting nutrient absorption (3). However, in recent years, concerns have arisen regarding the emergence of antibiotic resistance genes due to the utilization of antibiotics, which poses a potential threat to human health (4). In 2020, antibiotics were prohibited for use as growth promoters in feed by the Chinese Ministry of Agriculture and Rural Affairs. Therefore, alleviating stress damage caused by weaning to maintain piglet health is a pressing problem that needs to be solved. Owing to their natural properties, versatility, minimal toxicity, and low risk of resistance development, natural plant extracts demonstrate promising potential as alternative antibiotics (5). Numerous studies have shown that plant extracts possess immune-enhancing (6, 7), anti-inflammatory (8, 9), and antioxidant (10) effects in animals.

*Astragalus membranaceus* (AS), derived from dried roots of *Astragalus membranaceus* (Fisch.) Bge. var. mongholicus (Bge.) Hsiao is one of the most frequently used traditional Chinese herbal medicines. AS extracts have been demonstrated to exert antioxidant (11), anti-inflammatory (12), immune-modulating (13, 14), and antitumor and anticancer (15) effects in mouse models and humans. More than 100 compounds have been extracted from AS so far, with saponins, flavonoids, and polysaccharides being the primary constituents (16). Because of the simple extraction process and easy access, the beneficial functions of AS polysaccharides have been widely demonstrated. In recent years, increasing research on Astragaloside IV has confirmed its anti-oxidative, anti-inflammatory, anti-apoptotic, and immune-enhancing effects (17, 18).

Flavonoids are key active compounds in AS. Their antioxidant, antimicrobial, anti-inflammatory, immune-regulatory, and anti-tumor activities have been widely identified (19–21). Calycosin (CA) is an important flavonoid in AS. The glycoside form of CA, CA-7-glucoside, was described in the Chinese Pharmacopoeia a quality control index component of AS in 2010. Several clinical studies have elucidated the diverse pharmacological properties of CA, including anti-inflammatory, antioxidant, anti-osteoporosis, anticancer, and neuroprotective effects (22–25). Our previous study found that CA triggered the production of host defense peptides (HDPs) in IPEC-J2 cells (26). However, there is currently a lack of reports on the effects of CA on the growth performance and intestinal health of weaned piglets. Therefore, based on our previous study, this investigation systematically assessed the effects of AS CA and AS total flavone (TF) on the growth performance, immune function, and intestinal health of weaned piglets, aimed to establish a theoretical framework for the utilization of AS CA and AS TF extracts in swine husbandry.

## 2 Materials and methods

All animals involved in the present study were strictly managed in line with the constitution of the Experimental Animal Ethics Committee of Gansu Agricultural University (approval No. GSAU-Eth-AST-2023-006), international animal welfare and ethical standards, and relevant laws, regulations, and policies on the management of laboratory animals in Gansu Province.

### 2.1 Animals and experimental design

Sixty-four weaned piglets [Duroc × (Landrace × Yorkshire)] were assigned 4 treatment groups (initial average body weight of 8.12 ± 0.11 kg), each with 4 replicates comprising 4 piglets per replicate (2 females and 2 males): (1) CON: the basal diet, (2) P-CON: the basal diet plus 1 g/kg antibiotics (25% chlortetracycline premix), (3) AS-TF: the basal diet plus AS total flavone product (purity > 50%; consists of calycosin-7-glucoside, calycosin, formononetin-7-o-β-d-glucopyranoside, and formononetin) at 60 mg/day per piglet, and (4) AS-CA: the basal diet plus AS calycosin product (purity 20- 30%) at 30 mg/day per piglet. TF and CA were added to the basal diet by step-by-step mixing. AS TF and CA products were purchased from Chengdu KingTiger and Pharmaceutical Chemical Technology Co., Ltd. Chlortetracycline premix (25%) from Zhumadian Huazhong Chiatal. The basal diet was formulated following the piglet nutritional recommendations established by the National Research Council (2012), and the ingredient composition and nutrient levels of the basal diet are detailed in **Table 1**. The acclimatization period spanned 5 days, followed by a 14-day experiment. The feeding trial was conducted at Liuhe Ecological Agriculture and Animal Husbandry, Zhangye, China, where no chronic health issues were observed in the herd throughout the experiment. Piglets were allowed unrestricted access to water and their respective diets. The ambient conditions in the feeding facility were maintained at 50-60% humidity and a temperature of 25-28°C.

**Table 1:** Ingredient composition and nutrient levels in the basal diet (on as-fed basis).

Item	Content,%
Ingredients	
Corn	59.46
Soybean meal	21.18
Extruded soybean	6.00
Whey powder	2.00
Fish meal	2.62
Wheat bran	2.50
Calcium hydrophosphate	1.19
L-Lys · HCl	0.80
DL-Met	0.25
Premix^1^	4
Total	100
Nutrient levels^2^	
Dry matter	89.89
Crud protein	18.67
Ether extract	3.08
Ash	5.06
Calcium	0.72
Total phosphorus	0.60
Digestible energy, MJ/kg	13.36
Methionine + cysteine	0.66
Lysine	1.03
Threonine	0.74
Tryptophan	0.22

^1^The premix provides the following per kilogram of diet: vitamin A 13,500 IU, vitamin D3 3,500 IU, vitamin E 45 IU, vitamin K3 13 mg, vitamin B1 35 mg, vitamin B2 30 mg, vitamin B6 40 mg, vitamin B12 200 μg, nicotinic acid 60 mg, folic acid 2.4 mg, biotin 0.27 mg, D-pantothenic acid 24 mg; Cu (CuSO4·5H2O) 30 mg, Fe (FeSO4·H2O) 250 mg, Mn (MnSO4·5H2O) 40 mg, Zn (ZnCl_2_·5H2O) 110 mg, Se (Na2SeO3·H2O) 0.75 mg, I (Ca(IO_3_) _2_) 0.8 mg, NaCl 20 g.

^2^Crude protein, ether extract, ash, calcium, total phosphorus, and dry matter were analyzed values. Other nutrient levels are calculated values according to NRC (2012).

### 2.2 Growth performance, diarrhea incidence, and relative organ weight

Daily feed intake for each pen is monitored daily, and BW was measured on days 1 and 14 after a 12-hour fasting period to determine the average daily feed intake (ADFI), average daily gain (ADG), and feed conversion ratio (FCR).

Diarrhea was monitored through visual assessment. At 09:00 and 16:00 h on days 1-14, feces were scored, consistently by the same person, as follows: 0: diarrhea, i.e., liquid stool that can be poured, 1: sloppy feces, i.e., unformed stool conforming to the shape of the container, 2: normal feces, i.e., formed and moist stool retaining its shape, and 3: well-formed feces, i.e., the stool remains both solid and soft (27). Scores were recorded at the pen level by closely monitoring the behavior of individual pigs and assessing the overall signs of stool consistency within each pen, determined by calculating the average value of 4 pigs per pen. For each replicate (pen), the diarrhea rate = cumulative number of piglets with diarrhea/number of days counted/number of piglets per replicate × 100%.

### 2.3 Nutrient digestibility

Three days before the end of the trial, we collected the feces from each pen and then mixed it. The feces were soaked in 20% sulfuric acid, dried at 65°C, and stored at -20°C for future analysis of nutrient digestibility. The method described by Shi et al. (28) was utilized to determine the apparent total tract digestibility (ATTD) of nutrients. The acid-insoluble ash (AIA) concentrations in both diets and feces were determined following the methodology outlined by Prawirodigdo et al. (29). The levels of crude protein (CP, method 990.03), ether extract (EE, method 920.39), ash (method 961.14), and dry matter (DM, method 930.15) in diets and fecal samples were measured following the protocols outlined by the Association of Official Analytical Chemists (AOAC), 2007. ATTD of all nutrients was determined using the following formula:


$$\text{ATTD }(\%)=1-100\;\times \;\frac{\text{feces nutrient }\times \text{ diet AIA}}{\text{diet nutrient }\times \text{ feces AIA}}$$

### 2.4 Serum biochemistry and immunoglobulin and cytokine levels

On the 14th day, we collected a 10 mL blood specimen from the anterior vena cava of one piglet per replicate, whose BW closely matched the pen’s average. The collected blood was subsequently centrifuged at 3000 rpm at 4°C for 10 min. The serum was analyzed for levels of alanine aminotransferase (ALT), aspartate aminotransferase (AST), blood urea nitrogen (BUN), triglyceride (TG), total cholesterol (TC), total protein (TP), albumin (ALB), high-density lipoprotein cholesterol (HDL-C), and low-density lipoprotein cholesterol (LDL-C) using an automatic biochemical analyzer (Chemray 800, Servicebio Technology, Wuhan, China). The analyzer measured transmittance or absorbance using specific wavelengths and then calculated the concentrations of the target substances. The level of serum IgA, IgG, IgM, tumor necrosis factor α (TNF-α), and interleukin (IL) 1β, IL-6, IL-10, and intestinal mucosal secretory (s) IgA was measured using commercial ELISA kits according to the protocols provided by the manufacturer (Shanghai mlbio, Shanghai, China).

### 2.5 Intestinal morphology and goblet cell numbers

After blood collection, the piglets were electrocuted to death to collect tissues. Approximately 2 cm segments of the ileum and jejunum were excised and immersed in a 4% paraformaldehyde fixative solution, embedded in paraffin, and sectioned at 3-5 mm for subsequent hematoxylin-eosin (H&E) and periodic acid-Schiff (PAS) staining. Sections were stained with H&E to measure intestinal morphology [villus height (VH) and crypt depth (CD)]. Goblet cell numbers were determined by PAS staining, as outlined by Zhou et al. (30).

### 2.6 Reverse transcription-quantitative polymerase chain reaction

Total RNA was isolated from jejunal and ileal samples using the RNA-zol (Molecular Research Center, OH, USA). RNA concentrations were assessed with a DS-11 ultra-microphotometer (DeNovix, USA). cDNA was synthesized from the extracted RNA using the iScript cDNA synthesis kit (Bio-Rad, Hercules, CA, USA). qPCRs were performed using iTap Universal SYBR Green Supermix (Bio-Rad) in 10 μL reaction mixtures (5 μL SYBR, 0.3 μL of each of forward and reverse primers, 0.4 μL DEPC water, and 4 μL cDNA). Primers of *IL-12*, *TLR4*, *TGF-β*, *NF-κB*, *pBD129*, *PG 1-5*, and *pBD114* for qPCR were self-designed at NCBI, and *IL-1β*, *IL-8*, *TNF-α*, *pBD2*, *pBD3*, *pEP2C* and *GAPDH* were derived from previous studies (26) (**Table 2**). The annealing temperature of all primers is 60°C. Relative target gene expression was normalized to *GAPDH* expression and calculated using the 2−^△△Ct^ method.

**Table 2:** Primers are required for real-time fluorescence quantitative PCR.

Genes	Primer (5′→3′)	Product size, bp	Accession number
*IL-1β*	F: GCCCTGTACCCCAACTGGTA	61	NM_001302388.2
	R: CCAGGAAGACGGGCTTTTG		
*IL-8*	F: TTCGATGCCAGTGCATAAATA	176	NM_213867.1
	R: CTGTACAACCTTCTGCACCCA		
*IL-12*	F: AGGCCGTCAGCAACAC	160	NM_001354583.2
	R: GCAGCCAGGCAACTCT		
*NF-κB*	F: GTGTGTAAAGAAGCGGGACC	86	NM_001114281.1
	R: GCTCTTCTATGGGAACTTGGA		
*TNF-α*	F: CCCCTCTGAAAAAGACACCA	180	NM_214022.1
	R: TCGAAGTGCAGTAGGCAGAA		
*TLR-4*	F: TGCTTTCTCCGGGTCACTTC	203	NM_052892.5
	R: ATGTGGGGATGTTGTCAGGG		
*TGF-β*	F: TGTCTGTCCACCATTCATTTG	77	XM_021093503
	R: CACCAGGAGTACCTGCTCAAG		
*pBD1*	F: TTCCTCCTCATGGTCCTGTT	130	NM_213838.1
	R: AGGTGCCGATCTGTTTCATC		
*pBD2*	F: TGTCTGCCTCCTCTCTTCC	149	NM_214442.2
	R: AACAGGTCCCTTCAATCCTG		
*pBD3*	F: CCTTCTCTTTGCCTTGCTCTT	163	XM_021074698.1
	R: GCCACTCACAGAACAGCTACC		
*PG1-5*	F: GTAGGTTCTGCGTCTGTGTCG	196	XM_021070622.1
	R: CAAATCCTTCACCGTCTACCA		
*pEP2C*	F: ACTGCTTGTTCTCCAGAGCC	92	XM_003362076.4
	R: TGGCACAGATGACAAAGCCT		
*pBD129*	F: TCCGCACACTTGAAGAGGTC	121	NM_001129975.1
	R: CTGGCGAAAGGGTTGGTACT		
*pBD114*	F: TGGATCCTGAACGATGCTCAA	181	NM_001129973.1
	R: CTGGTGCACACATTGCATCT		
*GAPDH*	F: GCTACACTGAGGACCAGGTTG	146	XM_021091114.1
	R: CCTGTTGCTGTAGCCAAATTC		

### 2.7 16S rRNA gene sequencing

Jejunal digesta microbial total DNA was extracted using a MagAttract PowerSoil Pro DNA kit (Qiagn, Hilden, Germany). DNA quality was validated using a NanoDrop ND-2000 spectrophotometer (Thermo Scientific, USA). The V3-V4 region was amplified by PCR (Forward 5′- ACTCCTACGGGAGGCAGCA-3′; Reverse 5′- GGACTACHVGGGTWTCTAAT-3′), and then amplicons were sequenced on the Illumina MiSeq platform (Illumina, San Diego, USA). High-quality sequences were filtered using FASTP and denoised using the DADA2 plugin in QIIME2, generating amplicon sequence variants (ASVs). Based on the ASVs, rarefaction curves and α diversity indices were calculated with Mothur v1.30.2. The similarity among the microbial communities in different samples was determined by principal coordinate analysis (PCoA) based on Bray-curtis dissimilarity using the Vegan v2.4.3 package. The linear discriminant analysis (LDA) effect size (LEfSe) was performed to identify the significantly abundant taxa (phylum to genera) of bacteria among the different groups (LDA score > 2, *P* < 0.05). Further analyses were performed using the Majorbio Cloud platform (https://cloud.majorbio.com) to complete data visualization.

### 2.8 Statistical analysis

Dates were all presented as mean ± standard error of the mean (SEM). Using one-way analysis of variance (ANOVA) to analyze the difference after testing for normal distribution in the SPSS 26.0, and treatment comparisons were done using the LSD method for multiple testing. Significance was considered at *P* < 0.05, with 0.05 ≤ *P* < 0.10 indicating a tendency. Plots were generated using GraphPad Prism 8 (version 8.0.2, CA, USA).

## 3 Results

### 3.1 Growth performance, diarrhea incidence, and relative organ weights

As shown in **Table 3**, compared to the CON group, the final body weight (FBW), ADG, and ADFI of piglets significantly increased in the P-CON, AS-TF, and AS-CA groups. Moreover, P-CON and AS-CA significantly decreased the diarrhea rate and increased the fecal score of piglets compared to the CON group (*P* < 0.05), while the diarrhea rate in the AS-TF group showed a decreasing trend (*P* = 0.074). There were no significant differences in relative organ weights among all groups (*P* > 0.05, **Table 4**). In summary, AS-TF and AS-CA exert a positive effect on growth performance, and the effect is comparable to that of antibiotics.

**Table 3:** Effects of AS flavonoids on growth performance and diarrhea incidence in weaned piglets.

Item	CON	P-CON	AS- TF	AS-CA	SEM	*P*-Value
IBW, kg	8.13	8.10	8.11	8.13	0.028	0.990
FBW, kg	11.93^c^	13.42^a^	12.65^b^	13.06^ab^	0.150	0.004
ADG, g	261.25^b^	350.54^a^	329.22^a^	341.07^a^	9.99	<0.001
ADFI, g	449.40^c^	573.22^a^	520.84^b^	558.93^a^	12.87	<0.001
FCR	1.73	1.64	1.60	1.64	0.03	0.456
Diarrhea rate, %	2.68^a^	0.90^b^	1.34^ab^	0.45^b^	0.31	0.035
Fecal score	2.44^b^	2.92^a^	2.56^ab^	2.90^a^	0.08	0.032

IBW, initial body weight; FBW, final body weight; ADG, average daily gain; ADFI, average daily feed intake; FCR, feed conversion rate; CON, the basal diet; P-CON, the basal diet plus 1 g/kg antibiotics; AS-TF, the basal diet plus AS total flavone at 60 mg/day per piglet; AS-CA, the basal diet plus AS calycosin at 30 mg/day per piglet; SEM, standard error of the mean.

^a, b, c^Within a row, values with no common superscripts differ significantly (*P* < 0.05) between piglet feeding additives.

**Table 4:** Effects of AS flavonoids on relative organ weights in weaned piglets.

Item	CON	P-CON	AS- TF	AS-CA	SEM	*P*-Value
Spleen, g/kg	1.95	2.12	2.07	1.94	0.048	0.496
Liver, g/kg	26.33	29.19	28.08	26.32	0.633	0.314
Kidney, g/kg	4.92	5.08	5.03	5.09	0.114	0.963

CON, the basal diet; P-CON, the basal diet plus 1 g/kg antibiotics; AS-TF, the basal diet plus AS total flavone at 60 mg/day per piglet; AS-CA, the basal diet plus AS calycosin at 30 mg/day per piglet; SEM, standard error of the mean.

### 3.2 Nutrient apparent digestibility

As shown in **Table 5**, CP digestibility was higher in the AS-TF and AS-CA groups than in the CON group (*P* < 0.05). There were no significant differences in the apparent digestibility of DM and EE among all groups (*P* > 0.05).

**Table 5:** Effect of AS flavonoids on apparent digestibility of dietary nutrients in weaned piglets.

Item	CON	P-CON	AS- TF	AS-CA	SEM	*P*-Value
DM, %	86.85	86.25	87.93	89.59	0.704	0.411
CP, %	73.33^b^	77.54^ab^	80.67^a^	81.11^a^	1.310	0.040
EE, %	54.97	62.07	56.31	67.63	2.569	0.298

DM, dry matter; CP, crude protein; EE, ether extract; CON, the basal diet; P-CON, the basal diet plus 1 g/kg antibiotics; AS-TF, the basal diet plus AS total flavone at 60 mg/day per piglet; AS-CA, the basal diet plus AS calycosin at 30 mg/day per piglet; SEM, standard error of the mean.

^a, b^Within a row, values with no common superscripts differ significantly (*P* < 0.05) between piglet feeding additives.

### 3.3 Serum biochemistry parameter, immunoglobulins, and cytokine levels

#### 3.3.1 Serum biochemistry indices

As shown in **Table 6**, compared to the CON group, serum ALB contents were significantly increased in the P-CON, AS-TF, and AS-CA groups (*P* < 0.05). In contrast, the BUN and TG levels showed a significant decrease in the P-CON and AS-CA groups compared to the CON group (*P* < 0.05), and the BUN Level showed a downward trend in the AS-TF group (*P* = 0.062). Moreover, the BUN level in the AS-CA group was significantly lower than in the AS-TF group (*P* = 0.036). LDL-C contents were significantly decreased in the P-CON, AS-TF, and AS-CA groups (*P* < 0.05). Treatment with AS-TF significantly increased the serum TP level (*P* < 0.05), while the P-CON and AS-CA groups showed upward trends (*P* = 0.070, *P* = 0.069) compared to the CON group. The content of ALP significantly decreased in group P-CON compared to the CON group (*P* < 0.05). We also found that TC was decreased in AS-TF compared to the CON group. However, there were no significant differences in the level of AST, ALT, TC, and HDL-C among all groups (*P* > 0.05).

**Table 6:** Effect of AS flavonoids on serum biochemistry of weaned piglets.

Item	CON	P-CON	AS-TF	AS-CA	SEM	*P*-Value
TP, g/L	40.77^b^	43.08^ab^	43.64^a^	43.09^ab^	0.472	0.032
ALB, g/L	27.73^b^	31.71^a^	30.31^a^	30.45^a^	0.520	0.017
BUN, mmol/L	1.30^a^	0.69^bc^	0.96^ab^	0.57^c^	0.096	0.007
ALP, U/L	30.47^a^	18.28^b^	25.38^ab^	25.74^ab^	1.966	0.036
AST, U/L	78.15	60.07	55.41	59.68	4.621	0.342
ALT, U/L	67.73	69.15	75.82	74.88	4.159	0.904
HDL-C, mmol/L	0.81	0.79	0.77	0.86	0.019	0.423
LDL-C, mmol/L	1.23^a^	0.92^b^	0.93^b^	0.99^b^	0.047	0.031
TG, mmol/L	0.35^a^	0.29^b^	0.32^ab^	0.30^b^	0.008	0.035
TC, mmol/L	1.98^a^	1.73^ab^	1.58^b^	1.87^ab^	0.060	0.019

TP, total protein; ALB, albumin; BUN, blood urea nitrogen; ALP, alkaline phosphatase; AST, aspartate aminotransferase; ALT, Alanine aminotransferase; HDL-C, high-density lipoprotein cholesterol; LDL-C, low-density lipoprotein cholesterol; TG, triglyceride; TC, total cholesterol. CON, the basal diet; P-CON, the basal diet plus 1 g/kg antibiotics; AS-TF, the basal diet plus AS total flavone at 60 mg/day per piglet; AS-CA, the basal diet plus AS calycosin at 30 mg/day per piglet; SEM, standard error of the mean.

^a, b, c^Within a row, values with no common superscripts differ significantly (*P* < 0.05) between piglet feeding additives.

#### 3.3.2 Serum Ig, cytokine, and intestinal mucosal sIg levels

**Figure 1:**
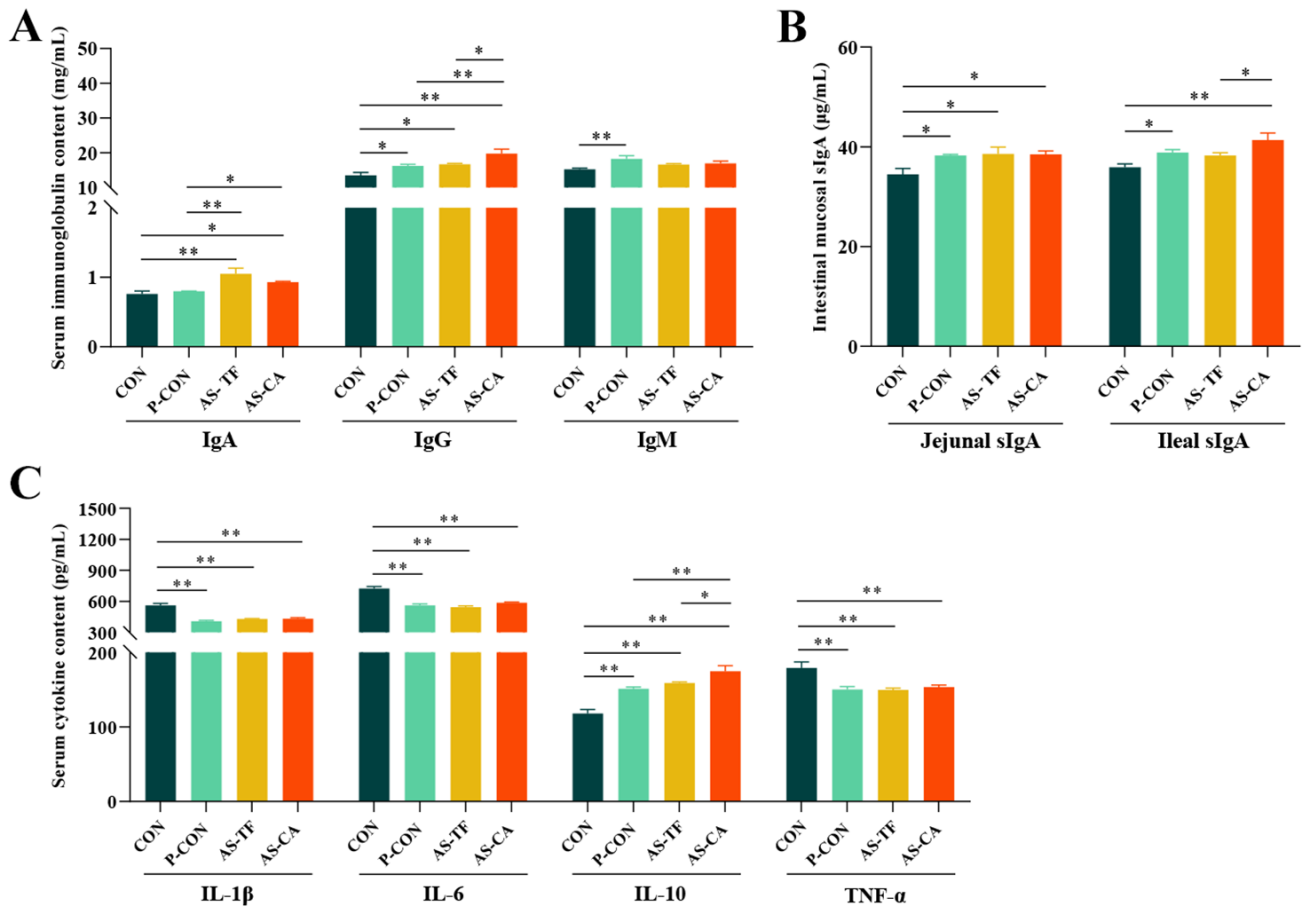
Effects of AS flavonoids on serum immunoglobulin (Ig) and cytokine and intestinal mucosal sIgA levels in weaned piglets. **(A)** Serum immunoglobulin A, G, M; **(B)** Jejunal and ileal mucosal sIgA. **(C)** Serum interleukin (IL) 1β, 6, 10, and tumor necrosis factor α (TNF-α). CON, the basal diet; P-CON, the basal diet plus 1 g/kg antibiotics; AS-TF, the basal diet plus AS total flavone at 60 mg/day per piglet; AS-CA, the basal diet plus AS calycosin at 30 mg/day per piglet. SEM, standard error of the mean. The data are expressed as mean ± SEM, n = 4 in each group. *P < 0.05, **P < 0.01.

Results of (**Figures 1A–C**) showed that serum IgA levels significantly increased in the AS-TF (*P* < 0.001) and AS-CA (*P* = 0.012) groups compared to the CON group. Serum IgG levels were significantly increased in the P-CON (*P* = 0.034), AS-TF (*P* = 0.017), and AS-CA (*P* < 0.001) groups, with AS-CA showing higher levels than P-CON (*P* = 0.009) and AS-TF (*P* = 0.019), whereas a significant increase in IgM was observed only in the P-CON group compared to the CON group (*P* = 0.004). Levels of serum IL-1β, IL-6, and TNF-α significantly reduced in the P-CON, AS-TF, and AS-CA groups (*P* < 0.01). Conversely, the level of serum IL-10 has a significant increase in the P-CON, AS-TF, and AS-CA groups, with the AS-CA group exhibiting higher levels than the AS-TF (*P* = 0.040) and P-CON (*P* = 0.005) groups. In addition, Jejunal sIgA levels were also significantly enhanced in all groups compared to the CON group (*P* < 0.05). The P-CON and AS-CA groups showed significant increases in the ileal sIgA level compared to the CON group (*P* < 0.05), and the effect of AS-CA was greater than that of AS-TF.

### 3.4 Intestinal morphology, goblet cell numbers

**Figure 2:**
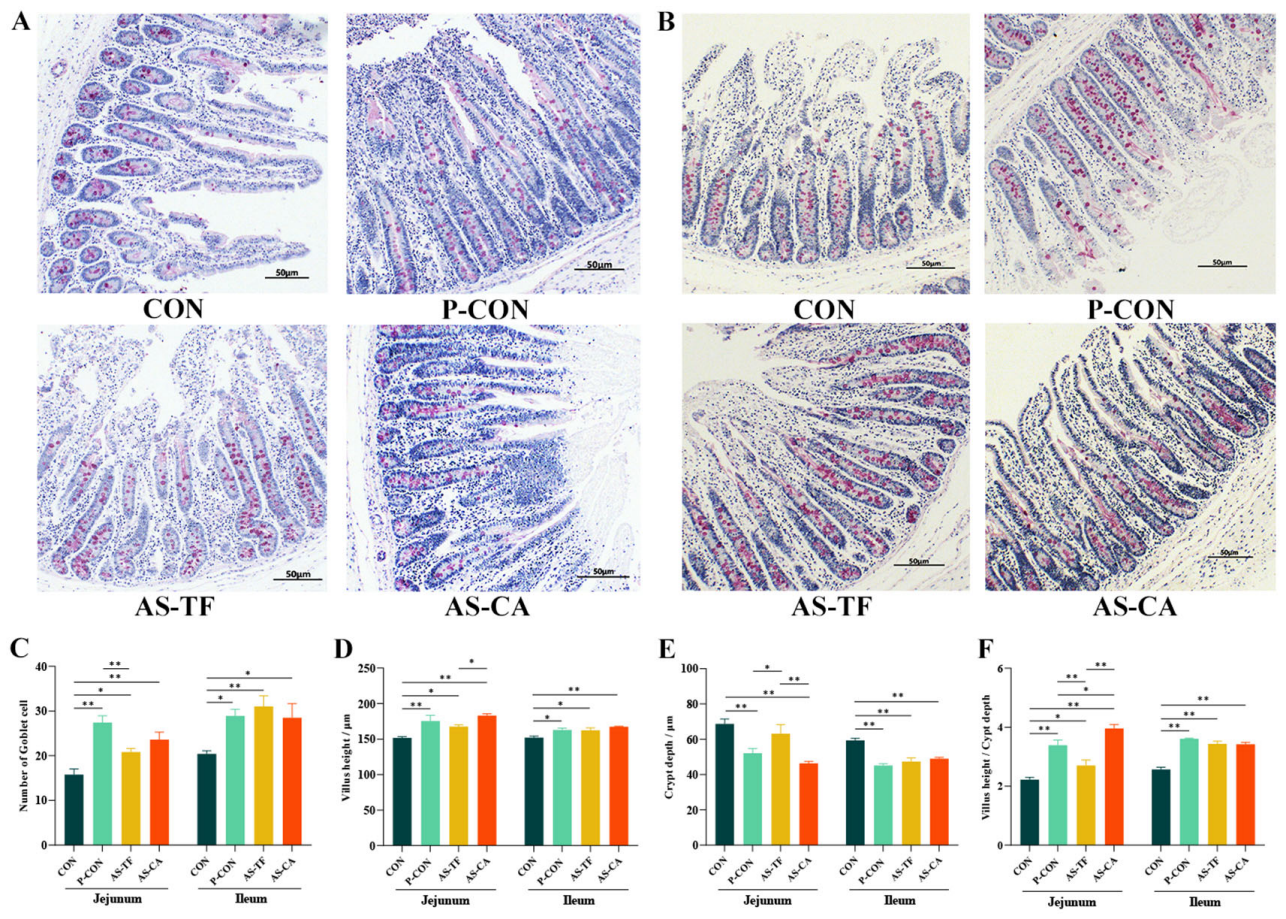
Jejunal and ileal morphology and numbers of goblet cells in weaned piglets. SEM, standard error of the mean. **(A, B)** Representative jejunal and ileal PAS staining images (40× magnification; scale bar = 50 μm). **(C)** Number of goblet cells in Jejunal and ileal. **(D)** Villus height of jejunal and ileal. **(E)** Crypt depth of jejunal and ileal. **(F)** Villus height/Crypt depth. CON, the basal diet; P-CON, the basal diet plus 1 g/kg antibiotics; AS-TF, the basal diet plus AS total flavone at 60 mg/day per piglet; AS-CA, the basal diet plus AS calycosin at 30 mg/day per piglet. The data are expressed as mean ± SEM, n = 4 in each group. *P < 0.05, **P < 0.01.

As illustrated in (**Figures 2A–C**), PAS staining results indicated a significant increase in goblet cell numbers in the jejunum in the P-CON, AS-TF, and AS-CA groups compared to the CON group (*P* < 0.05), with AS-TF being lower than P-CON (*P* < 0.05). Similarly, ileal goblet cell numbers were significantly increased in the P-CON, AS-TF, and AS-CA (*P* < 0.05) groups. H&E staining revealed a significant enhancement in the VH of the jejunum in the P-CON, AS-TF, and AS-CA (*P* < 0.05) groups compared to the CON group (*P* < 0.05), with AS-CA being more effective than AS-TF (*P* = 0.030). Ileal VH was also significantly improved in the three groups compared with the CON group (*P* < 0.05). Compared to the CON group, the jejunal CD was significantly decreased in the P-CON and AS-CA groups (*P* < 0.05), with AS-CA being more effective than AS-TF (*P* = 0.005), P-CON was better than AS-TF (*P* < 0.01). The ileal CD was significantly decreased in the P-CON, AS-TF, and AS-CA groups compared to the CON group (*P* < 0.001, **Figures 2D, E**). The ratio of jejunal and ileal VH/CD was significantly increased in the P-CON, AS-TF, and AS-CA groups compared to the CON group (*P* < 0.05, **Figure 2F**).

### 3.5 Intestinal inflammatory cytokine and HDP gene expression

**Figure 3:**
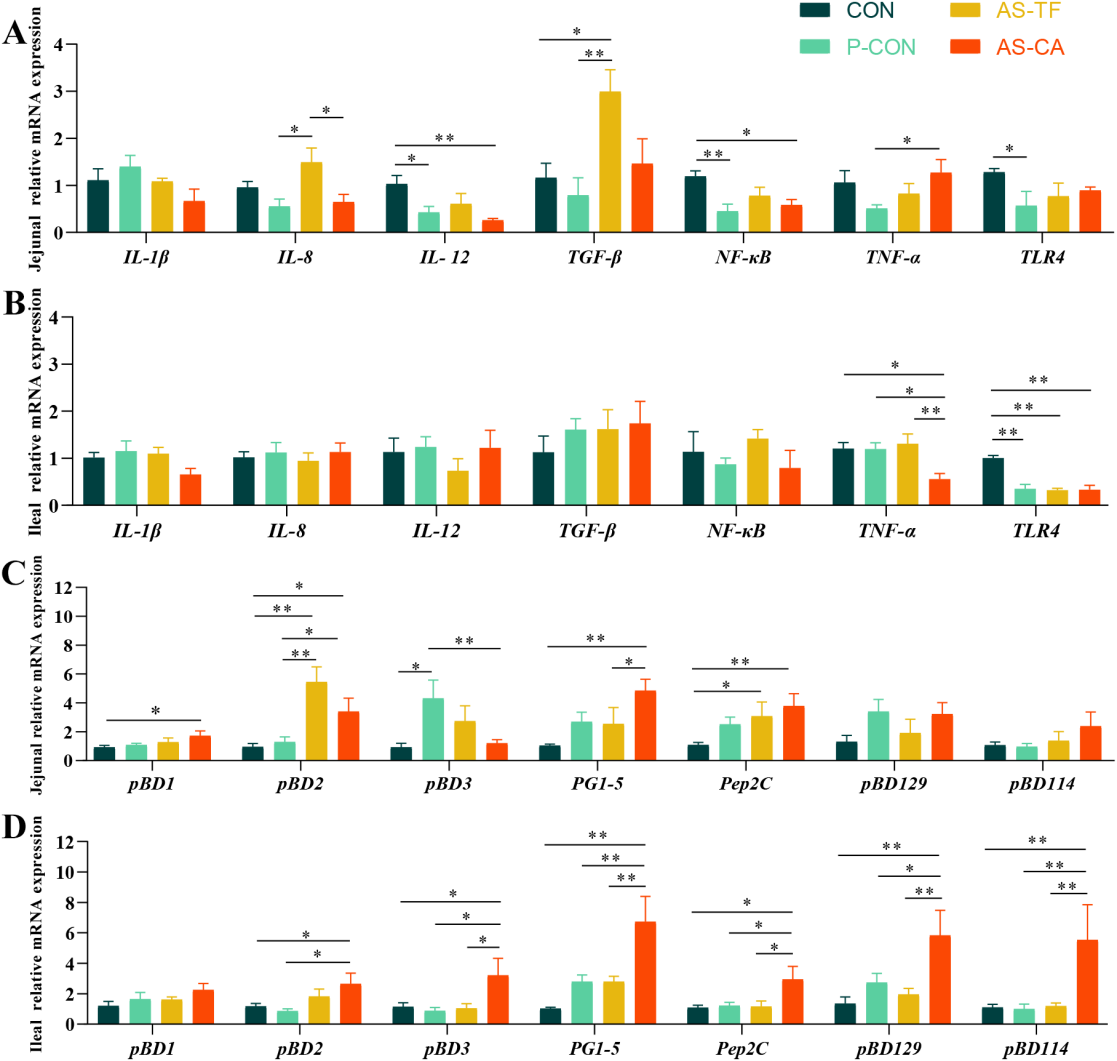
Effect of AS flavonoids on relative immune mRNA expression levels in the intestinal tract of weaned piglets. *TGF-β*, transforming growth factor-b; *NF-κB*, nuclear factor-κ-gene binding; *TLR-4*, toll like receptor-4; pBD-, porcine β-Defensin; PG-, protegrins; *pEP2C*, porcine epididymis protein 2 splicing variant C **(A)** Jejunal relative mRNA expression of cytokine. **(B)** Ileal relative mRNA expression cytokine. **(C)** Jejunal relative mRNA expression of HDP. **(D)** Ileal relative mRNA expression of HDP. CON, the basal diet; P-CON, the basal diet plus 1 g/kg antibiotics; AS-TF, the basal diet plus AS total flavone at 60 mg/day per piglet; AS-CA, the basal diet plus AS calycosin at 30 mg/day per piglet. The data are expressed as mean ± SEM, n = 4 in each group. *P < 0.05, **P < 0.01.

Compared to CON, both P-CON and AS-CA significantly reduced jejunal *IL-12* and *NF-κB* mRNA levels (*P* < 0.01, *P* < 0.05). P-CON suppressed *TLR-4* expression (*P* = 0.045) compared to CON, and AS-CA decreased the *IL-8* mRNA level (*P* < 0.05) compared to the AS-TF. Jejunal *TGF-β* expression was significantly upregulated in the AS-TF group compared with the CON and P-CON groups (*P* < 0.05, **Figure 3A**). Ileal *IL-1β*, *IL-8*, *IL-12*, *TGF-β*, and *NF-κB* expression showed no significant differences among all groups (*P* > 0.05). However, the AS-CA group significantly decreased the expression of *TNF-α* compared with other groups (*P* < 0.05). The *TLR-4* expression in P-CON, AS-TF, and AS-CA was also decreased compared to the CON group (*P* < 0.01, **Figure 3B**).

The effects of AS flavonoids on intestinal HDP gene expression are shown in (**Figures 3C, D**). In the jejunum, AS-CA significantly increased the expression of porcine β-defensin1 (*pBD1*), *pBD2*, protegrins 1-5 (*PG1-5*), and epididymis protein 2 splicing variant C (*pEP2C*) compared to CON group (*P* < 0.05), with *PG 1-5* expression being higher than those in the AS-TF group (*P* = 0.045). AS-CA also elevated *pBD2* (*P* = 0.035) expression compared to P-CON. AS-TF increased *pBD2* and *pEP2C* expression compared to the CON group (*P* < 0.05). In the ileum, AS-CA enhanced the expression of *pBD2*, *pBD3*, *PG1-5*, *pEP2C*, *pBD114*, and *pBD129* compared to CON and P-CON (*P* < 0.05). Furthermore, the expression of *pBD3*, *PG1-5*, *pEP2C*, *pBD114*, and *pBD129* in the AS-CA group were significantly higher than those in the AS-TF groups (*P* < 0.05). Overall, AS-CA showed a more pronounced effect than AS-TF in enhancing intestinal HDP gene expression, particularly in the ileum.

### 3.6 Jejunal microbiota

**Figure 4:**
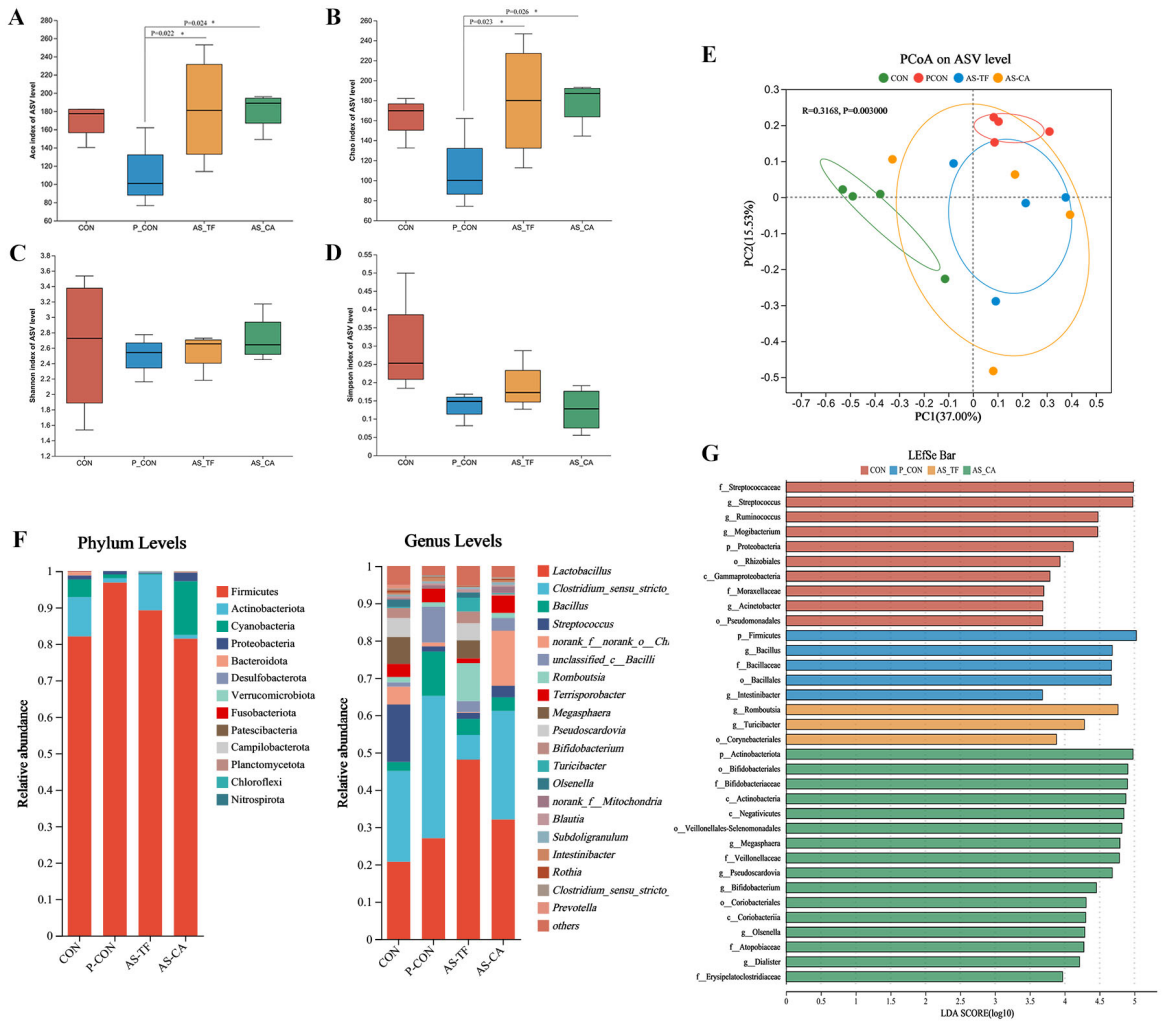
Effect of AS flavonoids on microbial composition and diversity in the jejunum of weaned piglets. **(A)** ACE index. **(B)** Chao index. **(C)** Shannon index. **(D)** Simpson index at the genus level. **(E)** Principal co-ordinates analysis. **(F)** Relative abundances at the phylum and genus levels. **(G)** Linear discriminant analysis effect size analysis. CON, the basal diet; P-CON, the basal diet plus 1 g/kg antibiotics; AS-TF, the basal diet plus AS total flavone at 60 mg/day per piglet; AS-CA, the basal diet plus AS calycosin at 30 mg/day per piglet. The data are expressed as mean ± SEM, n = 4 in each group. *P < 0.05.

A total of 357,600 high-quality reads were obtained from the sequencing of the 16S rRNA gene in 16 jejunal samples. As shown in (**Figures 4A–D**), the AS-TF and AS-CA treatments significantly increased the ACE and Chao index compared with the P-CON, but did not affect the Shannon and Simpson index. In PCoA performed at the ASV level, the jejunal samples of the P-CON and AS-TF groups showed clear clustering in terms of microbial community structure compared with CON (R = 0.3168, *P* = 0.003), with no differences among three test groups, indicating that antibiotics and AS flavonoids affect the gut microbiota composition and diversity in weaned piglets (**Figure 4E**). At the phylum levels, Firmicutes, Actinobacteria, Proteobacteria were relatively highly abundance in the jejunum of piglets in all groups. The relative abundances of Firmicutes in the P-CON group were significantly increased compared to those in the CON group (*P* < 0.05), whereas that of Proteobacteria was decreased. *Lactobacillus*, *Clostridium_sensu_stricto_1*, *Bacillus*, and *Streptococcus* were dominant in all jejunal microbiota samples in genus level (**Figure 4F**). LEfSe was used to identify significantly differential bacterial taxa from the phylum to genus levels between the CON group and test groups (**Figure 4G**). The LDA histogram reveals that 26 biomarkers had LDA scores > 4, with 5, 4, 2, and 15 dominant taxa in jejunal samples from the CON, P-CON, AS-TF, and AS-CA groups, respectively. *Streptococcus*, *Acinetobacter*, *Ruminococcus* and *Mogibacterium* were plentiful genera in the CON group, *Bacillus* and *Intestinibacter* were enriched in the P-CON group, *Romboutsia* and *Turicibacter* were enriched in the AS-TF group and *Megasphaera*, *Pseudoscardovia*, *Bifidobacterium*, and *Olsenella* were enriched in the AS-CA group.

### 3.7 Spearman correlation for serum cytokines and jejunal differential bacterial genera

**Figure 5:**
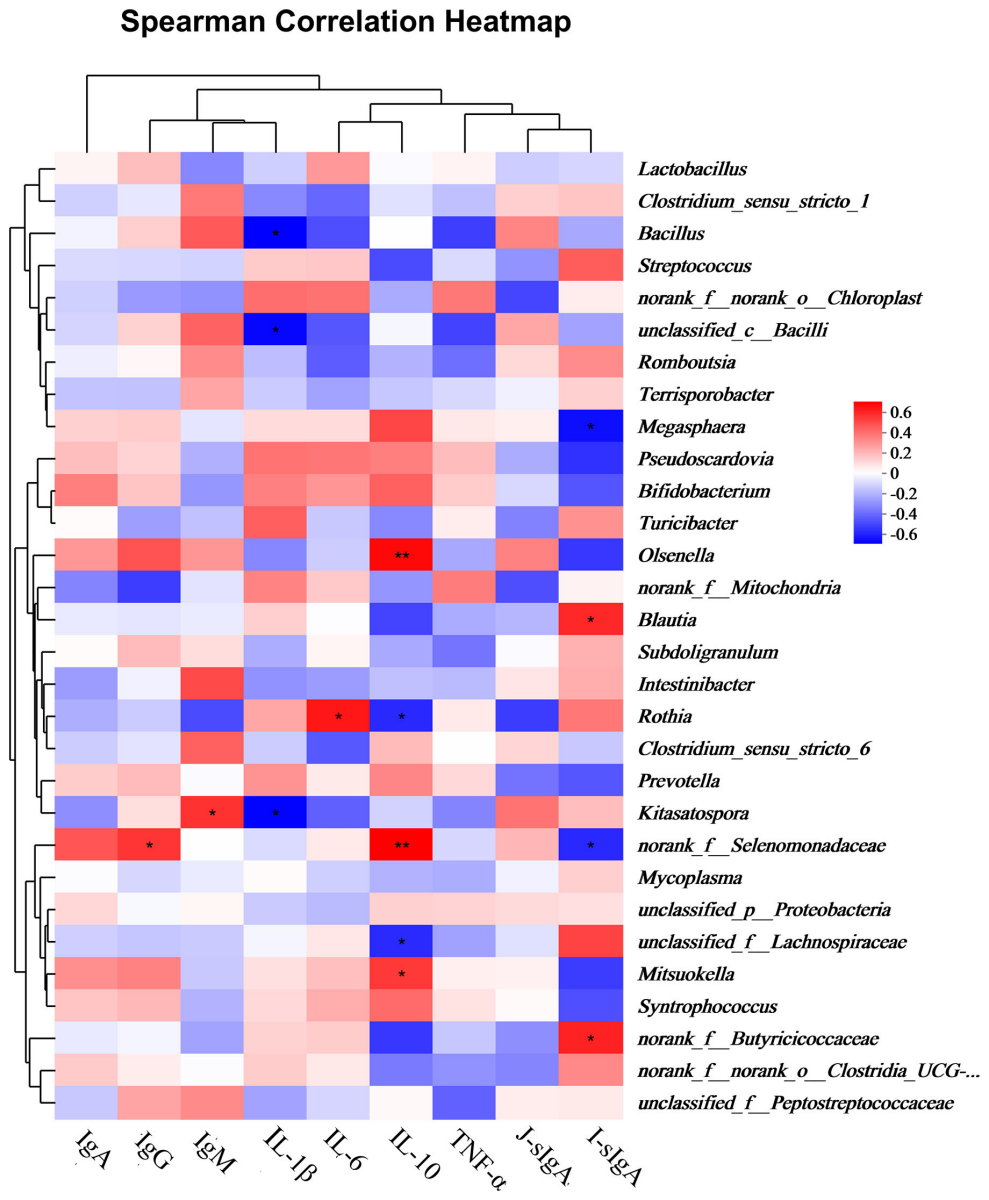
Spearman correlation heatmaps for serum cytokines and jejunal bacterial genera. IgA, IgG, IgM: serum immunoglobulins A, G, M; IL-1β, IL-6, IL-10: serum interleukins 1β, 6, 10; TNF-α: serum tumor necrosis factor α; J-sIgA, jejunal sIgA; I-sIgA: ileal sIgA. * 0.01 < *P* ≤ 0.05, ** 0.001 < *P* ≤ 0.01.

**Figure 5** shows the correlations between the differential jejunal bacterial genera and concentrations of serum Ig, cytokine, and intestinal mucosal sIg levels. Serum IgG and IgM levels were positively correlated with *norank_f:Selenomonadaceae* and *Kitasatospora*, respectively. The serum IL-1β level was negatively correlated with *Bacillus*, *unclassified_c:Bacilli*, and *Kitasatospora*. The serum IL-10 level was positively correlated with *Olsenella*, *norank_f:Selenomonadaceae*, and *Mitsuokella*, but negatively correlated with *Rothia* and *unclassified_f:Lachnospiraceae*. The ileal mucosal sIgA level was positively correlated with *Blautia* and *norank_f:Butyricicoccaceae*, and negatively correlated with *Megasphaera* and *norank_f:Selenomonadaceae*.

## 4 Discussion

Early weaning constitutes a critical approach for enhancing the efficiency of contemporary swine breeding systems; however, the sudden changes in the diet, feeding environment, and the reduction in maternal antibodies in piglets may result in inefficient use of nutritional resources, and susceptibility to pathogens, which can result in reduced production performance, diarrhea, and even mortality (5). Over the past few decades, antibiotics have been extensively employed to mitigate weaning stress and enhance the performance of weaned piglets. However, increasing evidence suggests that the abuse of antibiotics increases pathogen resistance and causes damage to the environment (1, 30). To alleviate weaning stress and improve growth efficiency and health, there is an urgent to explore natural, safe, and effective feed additives as antibiotic alternatives (31).

Flavonoids are major chemical components of Radix Astragali and include flavones, isoflavanes, flavonols, thiaxanthins, and chalcones (20, 32). They have demonstrated various medicinal benefits, such as anti-inflammatory, antioxidant, anticancer, and antiviral properties (33). Flavonoids have also shown beneficial effects in animal production. Under normal physiological conditions, supplementation of flavonoids from mulberry leaves and *Eucommia ulmoides* flavones (EUF) could increase the ADG and ADFI of growing pigs and weaned piglets, whereas EUF decreased the diarrhea index (34, 35). In the pathological state of weaned piglets challenged with diquat or deoxynivalenol, EUF also increased the growth performance and FCR of fatting pigs (36, 37). Baicalin extracted from *Radix Scutellaria* reduced mortality in piglets (38). In this study, the effect of AS flavonoids increased the growth performance, which is consistent with the above findings. Increased nutrient digestibility contributed to improved growth performance (39). The nutrient digestibility of CP, gross energy, and organic matter was improved by supplementation of tartary buckwheat flavonoids (40 mg/kg) in 35-day-old weaned piglets, and the ADG and FCR were also improved (40). We also found increased CP digestibility, suggesting that improved nutrient digestibility at least in part contributed to the increased growth performance in our study. Dietary inclusion of EUF significantly enhanced the BW, ADG, and FCR, while reducing the incidence of diarrhea in weanling piglets compared to antibiotics (35). In the current study, AS flavonoids exerted growth-promoting effects comparable to those of commercial antibiotics, which indicates that AS flavonoids as additives can replace antibiotics to enhance production performance. Further, we found that the effects of AS-CA on the growth performance and diarrhea rate of piglets were numerically superior to those of AS-TF. Therefore, we speculate that CA is a major beneficial chemical component in AS total flavone that contributes to piglet growth performance.

Serum concentrations of TP, ALB, and BUN reflect the absorption, synthesis, and decomposition of proteins (41, 42). Puerara flavonoids extracted from kudzu root, baicalin extracted from *Scutellaria baicalensis* Georgi, and *Eucommia ulmoides* flavones have been demonstrated to decrease serum BUN levels and enhance serum TP and ALB levels (35, 43, 44). Our results for these indices were consistent with these findings, which indicate that enhanced serum TP and ALB levels may increase the deposition of protein in the animal body, thereby improving nutrient absorption and growth (42). Licorice flavonoids increased the serum HDL-C concentration and reduced AST and ALP levels in weaned piglets (45). Similar results were observed in LDL-C levels and numerically reduced serum AST and ALP levels after feeding AS flavonoids. As a biomarker of hepatocyte damage, increased serum LDL-C, ALT, and ALP concentration may be caused by oxidative stress and inflammation in animals (46, 47). However, the relationship between serum biochemical indices and the health status of piglets requires further research.

The intestinal tract not only serves as the primary site for nutrient absorption but also is vital in the defense against external pathogens, comprising the body’s largest immune system (48). Weaning stress and diarrhea exacerbate intestinal injury and damage the morphological integrity of the intestinal tract (49). Dietary supplementation of *E. ulmoides* flavones and baicalin enhanced the VH and VH/CD in the jejunum and ileum of piglets (35, 43). We obtained similar results, indicating the protective effect of AS flavonoids on intestinal morphology. Goblet cells secrete mucin to create a mucosal barrier to protect epithelial cells from external damage (50). Wang et al. (26) found that total flavones of *Abelmoschus manihot* ameliorated the reduction in goblet cells caused by dextran sodium sulfate. In line herewith, an increase in goblet cell numbers was found in the present study. Intestinal structure integrity ensures efficient nutrient digestion and absorption and intestinal immune function. Improvements in small intestinal morphology may be part of the mechanism by which AS TF and AS CA improve growth performance (34, 51).

Inflammation is a defensive immune system reaction against local bacterial infections or injury partially regulated by cytokines (52). In weaning stress, the intestinal immune system is activated and produces large amounts of anti-inflammatory cytokines; however, excessive cytokine production may result in intestinal damage and dysfunction (34). Dietary supplementation of a citrus total flavonoid extract in lactating dairy cows linearly decreased serum TNF-α, IL-1β, IL-2, and IL-6 levels (53). Baicalin, a flavonoid from *S. baicalensis*, inhibited lipopolysaccharide-induced increases in *IL-1β*, *IL-18*, and *TNF-α* expression in porcine mononuclear phagocytes (54). Similar results were observed for both intestinal inflammatory cytokines expression and serum cytokine production, and the effect of AS-CA was superior to those of AS-TF and antibiotics, supposing that CA plays a major role in the effects of AS TF. As the main reactive substances of the humoral immune response, Ig levels can reflect the immune function of the body (55). Licorice flavonoid powder enhanced the production of serum Ig (45). Similarly, we detected elevated serum Ig and intestinal sIgA levels. Furthermore, HDP gene expression in the jejunum and ileum of weaned piglets was significantly increased by adding AS flavonoids and, particularly, AS-CA. HDPs are essential elements of the innate immune system and are vital for mucosal defense, and their induction can enhance intestinal epithelial cell function and host resistance to pathogens (56, 57). Our results indicate AS flavonoids may enhance immune function in weaning piglets by augmenting immune cell numbers, inflammatory cytokine production, and HDP expression.

The intestinal microbiome and its metabolites are essential for maintaining animal health. It has been suggested that plant extracts exert beneficial effects by modifying the intestinal microbiota composition (58). Flavonoids regulate the intestinal microbiota by promoting the growth of probiotics, inhibiting the proliferation of pathogenic bacteria, and increasing microbiota diversity, which is considered the major mechanism of flavonoid bioactivity *in vivo* (59–61). Dietary supplementation with quercetin altered the structure and increased the diversity of the gut microbiota compared to a basal diet (62). It was found that the microbial community richness changed after the addition of AS TF and CA compared to the antibiotics in this study. Similar results were reported by Cui et al. (40). Plant extracts can promote the proliferation of beneficial bacteria. Commercial citrus flavonoid extract increased the relative abundances of *Lactobacillus* and *Lachnospira* in weaned piglets (63). Baicalin protected intestinal integrity in rats by increasing the enriches of *Streptococcus* and *Bifidobacterium* (64). *Bifidobacterium* and *Romboutsia*, classified as physiologically advantageous bacteria, exert numerous vital physiological roles in the human body, including fortifying biological barriers, providing nutritional benefits, enhancing immune responses, and ameliorating gastrointestinal functionality (65) and they show high abundance in this study. We also found that *Olsenella*, *Romboutsia*, and *Mitsuokella* significantly correlated with serum anti-inflammatory cytokines and intestinal sIgA. It is demonstrated that *Olsenella* enhances immunotherapy responses via the production of the metabolite inosine (66). As a probiotic, *Mitsuokella* utilizes specific substrates to generate short-chain fatty acids which enhance gut health in piglets by promoting small intestinal cell growth and proliferation, intestinal barrier function, and reducing gut inflammation (67, 68). In addition, as an anaerobic bacterium with probiotic traits, *Blautia* is prevalent in mammalian intestines (69), we also found a significant correlation with ileal sIgA in weaned piglets in this study. Collectively, we speculate that dietary supplementation with AS TF and AS CA may modulate intestinal microbiota to alleviate intestinal inflammatory response induced by weaning stress, thereby improving the intestinal health and growth performance of weaned piglets.

## 5 Conclusion

In summary, this study demonstrated that dietary supplemented with AS TF and AS CA significantly alleviated intestinal structural dysfunction, improved immune function, and maintained intestinal homeostasis, thus contributing to improved growth performance and alleviated diarrhea in weaning piglets. Our results indicated that AS flavonoids may play a vital role in alleviating weaning stress in piglets and serve as a potential substitute for antibiotics, AS CA appears to be a major compound contributing to the effects of AS TF. However, further research is needed to determine the optimal dosage of AS CA and its underlying mechanism for regulating the immune function of weaned piglets.

## Data Availability

The datasets presented in this study can be found in online repositories. The names of the repository/repositories and accession number(s) can be found below: https://www.ncbi.nlm.nih.gov/, PRJNA1131148.
